# Cost of controlling the dengue vector *Aedes aegypti* in the Peruvian amazon

**DOI:** 10.17843/rpmesp.2024.411.12905

**Published:** 2024-03-27

**Authors:** Salomon Durand, Arles Paredes, Carlos Pacheco, Ray Fernandez, Jose Herrera, César Cabezas

**Affiliations:** 1 Instituto Peruano de Investigación en Salud, Lima, Peru Instituto Peruano de Investigaciones en Salud Lima Peru; 2 Regional Health Directorate of Loreto, Iquitos, Peru. Dirección Regional de salud Loreto Iquitos Peru; 3 Instituto Nacional de Salud, Lima, Peru. Instituto Nacional de Salud Lima Peru; 4 Universidad Nacional Mayor de San Marcos, Lima, Peru. Universidad Nacional Mayor de San Marcos Universidad Nacional Mayor de San Marcos Lima Peru

**Keywords:** Dengue, Aedes, Cost and Cost Analysis, Vector Control

## Abstract

**Objective.:**

To estimate the costs incurred in the control of *Aedes aegypti* in the Loreto region, during the years 2017 and 2018.

**Materials and methods.:**

We conducted a partial retrospective economic evaluation of the costs of *Aedes aegypti* control of the Regional Health Directorate Loreto, during the implementation of the Regional Plan for Surveillance and Control of *Aedes aegypti*. Documentation such as plans, intervention reports and payment slips were reviewed, and interviews were conducted with professional personnel involved in vector control, on the costs of control interventions.

**Results.:**

We found that the costs incurred in dengue vector control in the Loreto Region in the two years were: PEN 3,807,858 and PEN 4,066,380 during 2017 and 2018, respectively (USD 1,175,264 and USD 1,1210,232 at the 2017 and 2018 exchange rate). However, the effect of control activities is short-lived.

**Conclusions.:**

The high cost involved in vector control with the methods currently used and the short duration of its effect make it unsustainable. Studies should be conducted in order to find other more efficient methods for dengue control.

## INTRODUCTION

Since its reintroduction in 1990, dengue has become the most important vector-borne disease in Peru ^1^. Dengue is caused by the DENV arbovirus and transmitted by *Aedes aegypti* and *Aedes albopictus* (although the presence of the latter has not been reported in Peru), which cause outbreaks during the rainy season or when temperatures rise, collapsing health services, as occurred in Iquitos in 2011 after the entry of the Asian/American genotype of DENV-2 ^2^.

*Aedes aegypti*, since the first report of its reintroduction to Peru in 1984, has spread from the Amazon to almost the entire country; the mobility of people due to trade, migration or other reasons facilitates the involuntary transport of vector eggs in containers, infesting new localities ^3^. Twenty-one of Peru’s 24 departments have *Aedes aegypti* and 20 have dengue transmission ^3^. In the urban centers of the Amazon and in the coastal region, this disease is very prevalent and outbreaks are also currently being reported in small rural communities, which aggravates the problem ^1^.

In the 1950s, the elimination of this vector in Peru was possible within the framework of urban yellow fever eradication campaigns ^4^, but, at present, despite multiple efforts, this vector has not been controlled in a sustained manner ^1^.

The strategy used in Peru and other endemic countries to reduce the transmission of dengue is the control of its vector in its different stages. The main approach is the use of larvicides or inhibitors of its development in drinking water containers, the elimination of waste and the use of insecticides for adult mosquitoes, inside and outside homes. This strategy has been used for decades and its effectiveness and sustainability are being evaluated ^5^. In Peru, few studies have been published on the evaluation or application of new control methods ^6^. Likewise, the few studies on the evaluation of its costs do not describe the cost of vector control in a disaggregated manner ^7^^,^^8^.

In Peru, *Aedes aegypti* surveillance and vector control activities are funded by the state through regional governments, municipalities and the Ministry of Health (MINSA) ^9^. The Regional Health Directorate of Loreto (DIRESA Loreto), through the Environmental Health Directorate (DESA), carries out *Aedes aegypti* control interventions each year through two strategies: larval control, through entomological surveillance (aedic surveys), control with application of larvicide growth inhibitors, both through inspection of high-risk homes, and adult mosquito control through spatial fogging with portable equipment (Pulverizer motorcycles and thermo-nebulizers) according to MINSA regulations ^9^.

This study aimed to estimate the costs incurred in the control of *Aedes aegypti* in the Loreto region in 2017 and 2018.

**KEY MESSAGES**

Motivation for the study: Dengue prevention and control is based on the control of its vector. This study was conducted because of the need to know the costs associated with *Aedes aegypti* control in a region that carries out planned vector control activities.

Main findings: The costs incurred in dengue vector control in the Loreto region in 2017 and 2018 amounted to PEN 4,066,380.25 and PEN 3,807,858.73, respectively.

Implications: Knowing the cost of vector control activities will allow us to better plan these activities and have a basis for cost-effectiveness studies with other methods of prevention and control of dengue.

## MATERIALS AND METHODS

We conducted a descriptive cost study. The Loreto region has a population of 1,077,831 inhabitants, of which approximately 500,000 live in the city of Iquitos, located on the banks of the Amazon River. Iquitos has poor basic sanitation and water distribution by the hour, so families store water in containers such as cylinders, buckets and pots, which become potential breeding sites for the vector, also, high temperatures and constant rains favor infestation of homes ^6^.

This study was planned as a partial economic evaluation of intervention costs. Costs were estimated from the perspective of the public health system ^10^, considering that MINSA, under the Peruvian Dengue Surveillance and Vector Control Technical Standard ^9^, is the funder through DIRESA Loreto. All direct and indirect costs of the vector control program were included.

We analyzed dengue vector control interventions carried out from January 2017 to December 2018; for this purpose, the costs of interventions for both larval control and adult mosquito control, carried out in accordance with the current standard ^9^, including scheduled vector control or in response to outbreaks, were reviewed.

Data were collected on forms designed for this purpose, which compiled the information according to the classification of expenditures or costs (direct health, indirect and unit costs). All proposed dengue prevention, surveillance and control plans were requested from the DESA of the DIRESA. Then, documentation of all vector control interventions carried out during the two years of the study was requested, in compliance with the established plans and interventions in response to outbreaks; in addition to payment slips, receipts, payments, which were compared with the purchases and services entered into the Integrated Administrative Management System (SIGA). Interviews with the professional responsible for vector control and biologists directly involved in DESA’s *Aedes aegypti* surveillance and control activities were conducted to gather information on the plans and carried out activities. Logistics and human resources personnel were interviewed to obtain the costs of equipment and vehicle purchases, as well as the salaries of personnel working directly or indirectly in vector control interventions.

In order to estimate the cost of inputs, such as insecticides: malathion 57% emulsion concentrate (EC) (for adult mosquito control) and pyriproxyfen 0.5% (for control of the immature stages), we reviewed the database of MINSA’s National Center for the Supply of Strategic Health Resources (CENARES), which details the unit cost and annual acquisitions.

Costs were classified as proposed by Drummond *et al*. ^11^, who classify costs into health and non-health costs. Health costs are the costs related to the health intervention and its subsequent evolution and treatment, and are assumed by the health system. They include the time of health professionals, the price of supplies, personal protective equipment and sanitary products used, among others.

The direct health costs of the implementation of each intervention were collected according to each strategy carried out during field work, either from the annual periodic programming or in response to outbreaks. Plans and reports for each intervention (larval control or adult vector control) were evaluated. Direct health costs were divided into the costs of personnel directly involved in vector control interventions (brigade chief, fumigation operators, registrars, driver, etc.), support personnel also directly involved (vector control center director, administrative technicians, biologists, etc.), materials and supplies, and equipment and vehicles.

Indirect health costs were calculated from the salaries of DESA personnel who participate indirectly in vector control interventions, the cost of renting premises, mobilities, office expenses, security, etc. Only the cost of water and electricity for the main premises was considered, and not the other premises because they were not considered significant. For the cost of equipment and goods, the direct line depreciation was used at a rate of 10%, considering the calculated useful life of the equipment.

The information collected was analyzed using Microsoft Excel ® and SPSS 22 (IBM®). The costs were reported considering an average exchange rate in dollars of 3.2 PEN for both years (exchange rate reported by the Superintendency of Banking and Insurance of Peru for 2017 and 2018).

This study was approved by the Regional Directorate of Health Loreto (No. OS 0003344-2019) and was planned as a cost study of a mosquito control intervention, retrospective and with secondary data sources, so no review of the protocol by an ethics committee for human studies was considered necessary.

## RESULTS

For 2017, the cost of dengue vector control was PEN 3,807,858.73, while in 2018 it was PEN 4,066,380.25. Larval control in general caused higher cost with 2,562,881.50 PEN in 2017 and 2,239,406.50 PEN in 2018 (Table 1).

**Table 1:** Summary of total costs incurred in *Aedes aegypti* vector control interventions, Regional Health Directorate Loreto, 2017-2018.

Costs of dengue vector control	2018 (PEN)	2017 (PEN)
Adult mosquito control (fogging with portable equipment)	1,709,110.25	1,108,071.73
Larval control	2,239,406.50	2,562,881.50
Aedic surveys	117,863.50	134,888.50
Total	4,066,380.25	3,807,858.73

PEN: Peruvian soles.

Table 2 shows the costs of the interventions of the fogging campaigns with portable equipment for vector control of the adult stage of the mosquito. The costs are divided into direct health costs and indirect health costs. For 2017, only two interventions or fogging campaigns with portable equipment were carried out in the prioritized sectors of the city of Iquitos. Direct sanitary costs were divided into personnel who participated directly in the campaigns, with a total cost of PEN 599,040.00; support personnel, who worked in the vector control office with a total cost of PEN 57,140.00; then there are the inputs, protection and logistical materials with a total cost of PEN 301,393.90; within these is malathion 57% EC, with an annual cost of PEN 28,560.50 and fuels, which had a higher cost in this category. Finally, there are the costs of equipment, machinery and transportation, with an annual cost of 40,020.83 PEN. The total direct annual sanitary cost was 997,594.73 PEN, while the total indirect sanitary cost was 108,460.00 PEN.

**Table 2:** Annual cost of fumigation campaigns: direct and indirect health costs. Loreto Regional Health Directorate, 2017-2018.

			2017	2018	Total (PEN)
			Cost (PEN)	Cost (PEN)	
Direct sanitary costs					
	Fumigation intervention personnel				
		General supervisors - biologists	35,100.00	38,610.00	
		Brigade leaders	39,780.00	42,120.00	
		Fogging operators	397,800.00	421,200.00	
		Other	126,360.00	136,890.00	
		Total	599,040.00	638,820.00	1,237,860.00
	Support Staff				
		Responsible for vector control surveillance	6,720.00	10,080.00	
		Biologists	11,200.00	16,800.00	
		Administrative technician	3,240.00	4,860.00	
		Other	35,980.00	53,970.00	
		Total	57,140.00	85,710.00	142,850.00
	Supplies and materials				
		Petroleum	109,250.00	257,266.50	
		84 octane gasoline	75,000.00	23,940.00	
		Malathion 57% insecticide	28,560.50	44,454.00	
		Other	88,583.40	429,268.50	
		Total	301,393.90	754,929.00	1,056,322.90
	Equipment, machinery and transportation				
		Pulverizer motorcycle	20,187.50	30,281.25	
		Motorcycle trailer	2,666.67	4,150.00	
		Other	17,166.67	25,750.00	
		Total	40,020.83	60,181.25	
	Total direct health care costs		997,594.73	1,539,640.25	2,437,032.90
Indirect sanitary costs					
	DESA headquarters staff		Costo	Costo	
		Director	2,240.00	5,040.00	
		Administrator	1,280.00	2,880.00	
		Other	2,160.00	3,240.00	
		Total	5,680.00	11,160.00	16,840.00
	Office and miscellaneous expenses				
		Office supplies and equipment	2,500.00	3,750.00	
		Mobility rental (collective transport or buses)	96,000.00	144,000.00	
		Other	4,280.00	10,560.00	
		Total	102,780.00	158,310.00	261,090.00
	Total indirect sanitary costs		108,460.00	169,470.00	277,930.00
Total cost of mosquito control			1,106,054.73	1,709,110.25	2,714,962.90

PEN: Peruvian soles, DESA: Environmental Sanitation Directorate, DIRESA: Regional Health Directorate of Loreto.

During 2018, three fogging campaigns were carried out with portable equipment, in prioritized sectors of Iquitos. In terms of direct health costs, the annual cost for personnel who participated directly in the campaigns was 638,820.00 PEN; the annual cost for support personnel was 85,710.00 PEN; the annual cost for supplies, protection and logistical materials, was 754,929. 00 PEN. There was a large difference in this category compared to the previous year, due to the fact that more campaigns were conducted; in addition, in 2018, 90 octane gasoline were purchased and a larger amount of malathion was acquired, with an annual cost of 44,454.00 PEN. Equipment and transportation costs only reached an annual cost of PEN 60,181.25. Finally, the annual indirect sanitary cost was 169,470.00 PEN (Table 2).

Larval vector control is carried out in a programmed manner, in five campaigns or intervention cycles of two months each. These activities include surveillance activities such as aedic surveys, home inspections and the use of larval growth controllers or inhibitors, as well as the elimination of breeding sites and guidance on prevention in homes. There were 130 home inspectors, who were distributed in different establishments in the region, 108 in the city of Iquitos and 22 in the outskirts; these inspectors conducted surveys and applied piriproxifen 0.5% for larval control.

Table 3 shows the costs of the inspection of dwellings at risk of dengue transmission and the application of aedic surveys; this is divided into direct and indirect health costs. Direct health costs were divided into personnel who participated directly in the inspection of dwellings and the aedic survey, with a total cost for 2017 of 2,025,600.00 PEN, support staff working in the vector control office with a total cost of 83,160.00 PEN, inputs, protection and logistical materials with a total cost of 549,185.00 PEN, and within these is the insecticide piriproxifen 0.5% with a cost of 375,900.00 PEN. Finally, there were other expenses such as training with 7,225.00 PEN; and aedic surveys with 134,888.50 PEN.

**Table 3:** Annual cost of home inspections and aedic surveys: direct and indirect health costs. Loreto Regional Health Directorate, 2017 - 2018.

			2017	2018	Total (PEN)
			Cost (PEN)	Cost (PEN)	
Direct sanitary costs					
	Main professional staff				
		Brigade chiefs	580,800.00	660,000.00	1,240,800.00
		Inspectors	1,372,560.00	1,231,200.00	2,603,760.00
		Aedic surveys	72,240.00	64,800.00	137,040.00
		Total	2,025,600.00	1,956,000.00	3,981,600.00
	Support Staff				
		Biologists	60,000.00	60,000.00	
		Director of the vector control center	13,440.00	13,440.00	
		Administrative technician	9,720.00	9,720.00	
		Total	83,160.00	83,160.00	166,320.00
	Supplies and materials				
		Piripoxyfen 0.5%.	375,900.00	105,000.00	
		Gloves	39,000.00	39,000.00	
		Backpack	32,500.00	32,500.00	
		T-shirts	7,800.00	7,800.00	
		Other	31,336.50	42,751.50	
		Materials for aedic survey	62,648.50	51,433.50	
		Total	549,185.00	278,485.00	827,670.00
	Other expenses				
		Training	7,225.00	7,225.00	14,450.00
		Aedic surveys	134,888.50	116,233.50	251,122.00
	Total direct sanitary costs		2,665,170.00	2,324,870.00	4,990,040.00
Indirect sanitary costs					
	DESA headquarters staff				
		Chief Executive Officer	4,500.00	4,500.00	
		Administrator	3,500.00	3,500.00	
		Other	4,200.00	4,200.00	
		Total	12,200.00	12,200.00	24,400.00
	Office and miscellaneous expenses				
		Office supplies and equipment	5,000.00	5,000.00	
		DESA main premises for rent	11,000.00	11,000.00	
		Other	4,200.00	4,200.00	
		Total	20,200.00	20,200.00	40,400.00
	Total indirect sanitary costs		32,400.00	32,400.00	64,800.00
Total cost of larval control			2,697,570.00	2,357,270.00	5,054,840.00

PEN: Peruvian soles, DESA: Executive Directorate of Environmental Sanitation, DIRESA: Regional Health Directorate of Loreto.

In 2018, the cost of housing inspector personnel was PEN 1,956,000.00; the cost of support personnel was PEN 83,160.00; the cost of supplies, personal protection and logistical materials was PEN 278,485.00, this cost includes pyriproxyfen at 0.5% with a cost of PEN 105,000.00; with a substantial difference when compared to the previous year. Finally, other expenses include training for inspectors at a cost of PEN 7,225.00 and aedic surveys at PEN 116,233.50.

The indirect health costs, for both 2017 and 2018, were 32,400.00 PEN per year.

Dengue cases increased seasonally in the two years of study, as reported by the Directorate of Epidemiology of DIRESA Loreto.

## DISCUSSION

The total annual cost of *Aedes aegypti* control in the Loreto region was PEN 3,807,858.73 (USD 1,175,264) for 2017 and PEN 4,066,380.25 (USD 1,210,232) for 2018.

Vector control activities in Peru are carried out in accordance with MINSA regulations ^9^. Vector control is carried out in scenarios II and III (presence of the vector with sporadic cases and presence of the vector in outbreaks). In scenario II, the objective is to reduce the risk of dengue transmission and is aimed at controlling the mosquito in its larval stage. In scenario III, the objective is to rapidly control transmission, and mosquito control methods are applied in both the larval and adult phases.

Our results shows that costs were higher compared to those reported in other countries, considering that Loreto has about one million inhabitants; however, significant resources are devoted to vector control in other countries where dengue is transmitted, such as Peru ^12^^-^^15^. There are few studies on costs in Peru and these do not describe in detail the expenses incurred in vector control. In Piura, in 2002, an expenditure of 64,260.00 PEN in 50 days for vector control of a dengue outbreak was reported in the town of Sechura ^16^. Salmon-Mulanovich *et al*. in 2014, calculated the cost of case care, during a dengue outbreak in an Amazonian region, finding that the cost for each case was 105.3 USD on average ^7^. Stahl *et al*. estimated the cost of the 2011 outbreak in Peru at USD 4.5 million, of which 16% corresponded to vector control (USD 738,701) ^8^.

The vector control methods that were used would be effective in reducing vector density ^17^; programmed vector control (larval control, insecticides, biologicals, etc.) would cost less than vector control in response to outbreaks. In Peru, the effectiveness of vector control has been evaluated. Stoddard *et al*., in a study that recorded data for a decade in the city of Iquitos, concluded that there is evidence of the impact of vector control of adults on dengue transmission if applied early in outbreaks ^18^. On the other hand, in Iquitos, Reiner *et al*. elaborated a model based on the density of *Aedes aegypti* adults, collected over several years, which showed that, depending on the number of houses covered during fogging, a reduction in the density of female adults, responsible for transmission, can be achieved between 67 and 43% if 100 or 50% of the houses are covered during fogging, respectively ^19^.

The methods that were used would be effective in reducing vector density, but their effect is short-lived and more costly over time. According to Pontes *et al*. larval control would persist for only two months in the best case ^20^. Adult control with ultra-low volume insecticide application has an immediate effect on the mosquito population, which would last only one day, according to Koenraadt *et al.*^21^. Given the short effect of these methods, in order to maintain a low density of *Aedes aegypti*, it is necessary to periodically carry out larval control cycles and adult control, all at a high cost, not achieving an extended effect or definitive vector control with the techniques currently used. We must also bear in mind that, it is sometimes necessary to increase the frequency of interventions for adult control in outbreaks with a large affected population. In 2011, according to DIRESA Loreto entomologists, more than 10 interventions were made for adults in one year due to the magnitude of the outbreak. On the other hand, the effect of interventions based on insecticides decreases over time due to the emergence of vector resistance, having to replace them with more costly and toxic molecules ^1^.

For example, in Iquitos, as in other cities in the country, the water supply lasts for less than five hours a day, which forces the population to store water in all types of containers that become breeding places for the vector ^6^. Likewise, the accumulation of waste and frequent rains creates conditions for the vector to grow.

During 2017, one of the largest outbreaks of dengue fever recorded by MINSA in Peru occurred on the northern Peruvian coast, with 68,290 cases and 89 deaths ^3^. In the same year, there was only a seasonal increase in cases in Loreto, perhaps as an effect of systematic vector control or climate.

A recent phenomenon is the invasion of *Aedes aegypti* into small rural communities, probably due to habits that facilitate the development of the vector, which would increase the costs of control ^22^.

One limitation of this study was that the analysis of adult mosquito control (fogging with portable equipment) was based mainly on data from the city of Iquitos, because the interventions carried out in other cities of the Loreto Region are difficult to quantify due to underreporting. Likewise, water and electricity costs for other rented premises were not considered because they were not considered significant in comparison to other costs. However, this study may serve as a baseline for subsequent studies to evaluate the impact, taking into account that the poor quality of the data and the existing underreporting in endemic countries make it difficult to analyze the impact of vector control ^24^. We should consider that this estimate was made in years with a moderate number of cases and not in a year with intense transmission as occurred in 2011 in Loreto, which would have increased costs.

In conclusion, the costs incurred in dengue vector control in the Loreto Region during the two years studied exceed US$1 million per year. These interventions have the immediate effect of reducing transmission, but this effect is short-lived and creates a dependence on the use of increasingly expensive insecticides.
