# Supplementary material for: Cost of controlling the dengue vector Aedes aegypti in the Peruvian amazon
Source: Rev Peru Med Exp Salud Publica. 2024 Mar 27;41(1):46–53. doi: 10.17843/rpmesp.2024.411.12905 (PMC11149769; doi:10.17843/rpmesp.2024.411.12905)
Supplement: Translation

# Translation: Costo del control de *Aedes aegypti* en la Amazonía peruana

**Authors:** Salomon Durand, Arles Paredes, Carlos Pacheco, Ray Fernandez, Jose Herrera, César Cabezas

**Affiliations:** 1 Instituto Peruano de Investigación en Salud, Lima, Perú; 2 Dirección Regional de salud Loreto, Iquitos, Perú.; 3 Instituto Nacional de Salud, Lima, Perú.; 4 Universidad Nacional Mayor de San Marcos, Lima, Perú.

**Keywords:** Dengue, Aedes, Costos y Análisis de Costo, Control Vectorial

## Abstract

**Objetivo.:**

Estimar los costos incurridos en el control del *Aedes aegypti* en la región Loreto, en los años 2017 y 2018.

**Materiales y métodos.:**

Se realizó una evaluación económica retrospectiva parcial de los costos del control del *Aedes aegypti* de la Dirección Regional de Salud Loreto, durante la ejecución del Plan Regional de Vigilancia y Control de *Aedes aegypti*. Se revisó documentación como planes, informes de intervenciones y planillas de pago y se realizaron entrevistas al personal profesional implicado en el control vectorial, sobre los costos de las intervenciones de control.

**Resultados.:**

Se halló, que los costos incurridos en el control del vector del dengue en la Región Loreto en los dos años estudiados ascienden a: 3,807,858 PEN y 4,066,380 PEN durante el 2017 y 2018, respectivamente (1´175,264 USD y 1´1210,232 USD al tipo de cambio del 2017 y 2018). Sin embargo, el efecto de las actividades de control es de corta duración.

**Conclusiones.:**

El alto costo que implica el control vectorial con los métodos usados actualmente y la corta duración de su efecto lo hace insostenible. Se deben realizar estudios para hallar otros métodos más eficientes para el control del dengue.

## INTRODUCCIÓN

Desde su reintroducción en 1990, el dengue se convirtió en la enfermedad más importante transmitida por vectores en el Perú ^1^^).^ El dengue es causado por el arbovirus DENV y transmitido por el *Aedes aegypti* y *Aedes albopictus* (aunque en el Perú no se ha reportado la presencia de este último), que originan brotes en época de lluvias o de incremento de temperatura, colapsando los servicios de salud, como ocurrió en Iquitos en el 2011, luego del ingreso del genotipo asiático/americano del DENV-2 ^2^.

El *Aedes aegypti*, desde el primer reporte de su reintroducción al Perú en 1984, se dispersó desde la Amazonía a casi todo el país; la movilidad de las personas por comercio, migración u otros motivos facilita el transporte involuntario de los huevos del vector en recipientes, infestando nuevas localidades ^3^. Veintiuno de los 24 departamentos del Perú, tienen presencia de *Aedes aegypti* y 20 tienen transmisión del dengue ^3^. En los centros urbanos de la Amazonía y en la región de la costa esta enfermedad es muy prevalente y actualmente también se reportan brotes en pequeñas comunidades rurales, lo que agrava el problema ^1^.

En la década de los 50, la eliminación de este vector en el país fue posible en el marco de las campañas de erradicación de la fiebre amarilla urbana ^4^, pero, en la actualidad, a pesar de los múltiples esfuerzos no se consigue controlar de manera sostenida a este vector ^1^.

La estrategia usada en el Perú y en otros países endémicos para reducir la transmisión del dengue es el control de su vector, en sus diferentes estadios. El principal enfoque es el uso de larvicidas o inhibidores de su desarrollo en recipientes de recolección de agua para consumo, la eliminación de inservibles y el empleo de insecticidas para mosquitos adultos, dentro y fuera de las viviendas. Esta estrategia se ha utilizado por décadas y se está evaluando su efectividad y sostenibilidad ^5^. En el Perú, se han publicado pocos estudios sobre la evaluación o la aplicación de nuevas metodologías de control ^6^. Asimismo, los pocos trabajos sobre la evaluación de sus costos no describen el costo del control vectorial de manera desagregada ^7^^,^^8^.

En el Perú, las actividades de vigilancia y control vectorial del *Aedes aegypti* son financiadas por el estado a través de los gobiernos regionales, municipios y el Ministerio de Salud (MINSA) ^9^. La Dirección Regional de Salud Loreto (DIRESA Loreto), a través de la Dirección de Salud Ambiental (DESA), realiza cada año intervenciones de control del *Aedes aegypti* mediante dos estrategias: el control larvario, a través de la vigilancia entomológica (encuestas aédicas), control con aplicación de larvicidas inhibidores del crecimiento, ambos a través de la inspección de las viviendas con alto riesgo, y el control del mosquito adulto a través de la nebulización espacial con equipos portátiles (motopulverizadoras y termonebulizadoras) de acuerdo a lo normado por el MINSA ^9^.

El objetivo del estudio es estimar los costos incurridos en el control del *Aedes aegypti* en la región Loreto, en los años 2017 y 2018.

**MENSAJE CLAVE**

Motivación para realizar el estudio: La prevención y control del dengue se basa en el control de su vector. Este estudio se realizó por la necesidad de conocer los costos asociados al control *Aedes aegypti* en una región que realiza actividades planificadas de control vectorial.

Principales hallazgos: Los costos incurridos en el control del vector del dengue en la región Loreto en los años 2017 y 2018, ascienden a 4,066,380.25 y 3,807,858.73 PEN, respectivamente.

Implicancias: Conocer el costo de las actividades de control vectorial nos permitirá planificar mejor estas actividades y tener una base para estudios de costo efectividad con otros métodos de prevención y control del dengue.

## MATERIALES Y MÉTODOS

Se efectuó un estudio descriptivo de costos. La región Loreto, que tiene una población de 1 077 831 habitantes, de los que, aproximadamente 500 000 viven en la ciudad de Iquitos, ciudad ubicada al margen del río Amazonas. Iquitos tiene deficiente saneamiento básico, y distribución de agua por horas, por lo que las familias almacenan agua en recipientes como cilindros, baldes y ollas, que se convierten en potenciales criaderos del vector, asimismo, las altas temperaturas y las lluvias constantes favorecen la infestación de las viviendas ^6^.

Este estudio se planificó como una evaluación económica parcial de los costos de intervención. Los costos se estimaron desde la perspectiva del sistema de salud pública ^10^, considerando que el MINSA, en el marco de la Norma Técnica de Vigilancia y Control Vectorial del Dengue del Perú ^9^, es el financiador a través de la DIRESA Loreto. Se incluyeron todos los costos directos e indirectos del programa de control vectorial.

Se analizaron las intervenciones de control vectorial del dengue efectuadas desde enero del 2017 a diciembre del 2018; para lo cual se revisaron los costos de las intervenciones tanto del control larvario, como del control del mosquito adulto, realizados de acuerdo con la norma vigente ^9^, incluyendo el control vectorial programado o en respuesta a brotes.

Los datos se recolectaron en formularios diseñados para este fin, el cual recopiló la información según la clasificación de gastos o costos (sanitarios directos, indirectos y costos unitarios). Se solicitó a la DESA de la DIRESA todos los planes de prevención, vigilancia y control del dengue propuestos; luego se solicitó documentación de todas las intervenciones del control vectorial realizadas durante los dos años de estudio, en cumplimiento de la programación de los planes establecidos y las intervenciones en respuesta a brotes; además de planillas de pagos, boletas, pagos y se compararon con las compras y servicios ingresadas al Sistema Integrado de Gestión Administrativa (SIGA). También se llevaron a cabo entrevistas al profesional responsable de control vectorial y biólogos que participan directamente en las actividades de vigilancia y control de *Aedes aegypti* de la DESA para recopilar información de los planes y actividades realizadas. Se entrevistó a personal de logística y recursos humanos para adquirir los costos de compras de equipos y vehículos; además de sueldos del personal que trabaja directa o indirectamente en las intervenciones del control vectorial.

Para la estimación de costos de insumos, como los insecticidas: malatión 57% emulsión concentrada (EC) (para el control del mosquito adulto) y piriproxifen 0,5% (para el control en las fases inmaduras) se revisó la base de datos del Centro Nacional de Abastecimiento de Recursos Estratégicos en Salud (CENARES) del MINSA, donde se especifica el costo unitario y las adquisiciones anuales.

Los costos se clasificaron según lo propuesto por Drummond *et al*. ^11^, que clasifican los costos en sanitarios y no sanitarios. Los costos sanitarios son los costos relacionados con la intervención en salud y su posterior evolución y tratamiento, y son asumidos por el sistema de salud. Incluye el tiempo de los profesionales sanitarios; el precio de los insumos, equipos de protección personal y productos sanitarios utilizados, entre otros.

Los costos sanitarios directos de la implementación de cada intervención fueron recolectados de acuerdo con cada estrategia realizada durante el trabajo de campo, ya sea de la programación periódica anual o en respuesta a brotes. Se evaluaron los planes e informes de cada intervención realizada (control larvario o control de vector adulto). Los costos sanitarios directos se dividieron en costos del personal que participa directamente en las intervenciones de control vectorial (jefe de brigada, operarios de fumigación, registradores, chofer, personal para perifoneo, etc.); personal de apoyo que también participa directamente (director de centro de control vectorial, técnicos administrativos, biólogos, etc.); materiales e insumos, y equipos y vehículos.

En los costos sanitarios indirectos se calcularon de los sueldos del personal que trabaja en la DESA que participa indirectamente en las intervenciones de control vectorial, costos de alquiler de locales, movilidades, gastos de oficina, seguridad, etc. Se consideró solo el costo de agua y luz del local principal, y no los otros locales por considerarlos no significativos. Para el costo de los equipos y bienes se utilizó la depreciación de línea directa a una tasa del 10% considerando el tiempo de vida útil, calculado de estos equipos.

La información recopilada se analizó utilizando Microsoft Excel ® y SPSS 22 (IBM ®). Los costos se reportaron en soles (PEN), considerando como tipo de cambio promedio en dolares de 3,2 PEN para ambos años (tipo de cambio reportado por la Superintendencia de Banca y Seguros del Perú para el 2017 y 2018).

Este estudio fue aprobado por la Dirección Regional de Salud Loreto. (N.º OS 0003344-2019) y se planificó como un estudio de costos de una intervención para el control de mosquitos, retrospectivo y con fuentes de datos secundarios, por lo que no se consideró necesaria la revisión del protocolo por un comité de ética para estudios en humanos.

## RESULTADOS

Para el 2017, el costo del control vectorial del dengue fue de 3,807,858.73 PEN, mientras que en el 2018 fue de 4,066,380.25 PEN. El control larvario en general ocasionó mayor costo con 2,562,881.50 PEN en el 2017 y 2,239,406.50 PEN en el 2018 (Tabla 1).

**Tabla 1:** Resumen de los costos totales incurridos en las intervenciones de control vectorial del *Aedes aegypti,* Dirección Regional de Salud Loreto, 2017-2018.

Costos del control vectorial de dengue	2018 (PEN)	2017 (PEN)
Control de mosquito adulto (nebulización espacial con equipo portátil)	1,709,110.25	1,108,071.73
Control larvario	2,239,406.50	2,562,881.50
Encuestas aédicas	117,863.50	134,888.50
Total	4,066,380.25	3,807,858.73

PEN: soles peruanos.

En la tabla 2 se muestran los costos de las intervenciones de las campañas de nebulización espacial con equipo portátil para el control del vector en la etapa adulta del mosquito. Los costos están divididos en costos sanitarios directos y costos sanitarios indirectos. Para el 2017, se realizaron solo dos intervenciones o campañas de nebulización espacial con equipo portátil, en los sectores priorizados de la ciudad de Iquitos. En cuanto a los costos sanitarios directos, este se dividió en personal que participa directamente en las campañas, con un costo total de 599,040.00 PEN; personal de apoyo, que labora en la oficina de control vectorial con un costo total de 57,140.00 PEN; luego están los insumos, materiales de protección y logísticos con un costo total de 301,393.90 PEN; dentro de estos está el malatión 57% EC, con un costo anual de 28,560.50 PEN y los combustibles que tuvieron un mayor costo en este rubro. Por último, están los costos de equipos, maquinarias y transporte; con un costo anual de 40,020.83 PEN. El costo sanitario anual directo total fue de 997,594.73 PEN, mientas el costo sanitario indirecto total fue de 108,460.00 PEN.

**Tabla 2:** Costo anual de las campañas de fumigación: costos sanitarios directos e indirectos. Dirección Regional de Salud Loreto, 2017-2018.

			2017	2018	Total (PEN)
			Costo (PEN)	Costo (PEN)	
Costos sanitarios directos					
	Personal de intervención fumigación				
		Supervisores generales - biólogos	35,100.00	38,610.00	
		Jefes de brigadas	39,780.00	42,120.00	
		Operarios de la nebulización espacial	397,800.00	421,200.00	
		Otros	126,360.00	136,890.00	
		Total	599,040.00	638,820.00	1,237,860.00
	Personal de Apoyo				
		Responsable de vigilancia de control de vectores	6,720.00	10,080.00	
		Biólogos	11,200.00	16,800.00	
		Técnico administrativo	3,240.00	4,860.00	
		Otros	35,980.00	53,970.00	
		Total	57,140.00	85,710.00	142,850.00
	Insumos y materiales				
		Petróleo	109,250.00	257,266.50	
		Gasolina de 84 octanos	75,000.00	23,940.00	
		Insecticida malatión 57%	28,560.50	44,454.00	
		Otros	88,583.40	429,268.50	
		Total	301,393.90	754,929.00	1,056,322.90
	Equipos, maquinaria y transporte				
		Moto pulverizadora	20,187.50	30,281.25	
		Moto furgonetas	2,666.67	4,150.00	
		Otros	17,166.67	25,750.00	
		Total	40,020.83	60,181.25	
	Total de costos sanitarios directos		997,594.73	1,539,640.25	2,437,032.90
Costos sanitarios indirectos					
	Personal de sede central DESA		Costo	Costo	
		Director	2,240.00	5,040.00	
		Administrador	1,280.00	2,880.00	
		Otros	2,160.00	3,240.00	
		Total	5,680.00	11,160.00	16,840.00
	Gastos de oficina y gastos varios				
		Material y equipos de oficina	2,500.00	3,750.00	
		Alquiler de movilidad (colectivos o buses)	96,000.00	144,000.00	
		Otros	4,280.00	10,560.00	
		Total	102,780.00	158,310.00	261,090.00
	Total de costos sanitarios indirectos		108,460.00	169,470.00	277,930.00
Costo total del control de mosquito			1,106,054.73	1,709,110.25	2,714,962.90

PEN: soles peruanos, DESA: Dirección de saneamiento ambiental, DIRESA: Dirección Regional de Salud Loreto.

Durante el 2018 se realizaron tres campañas de nebulización espacial con equipo portátil, en sectores priorizados de Iquitos. En cuanto a los costos sanitarios directos, costo anual por concepto del personal que participó directamente en las campañas fue de 638,820.00 PEN; el costo anual por personal de apoyo fue de 85,710.00 PEN; el costo anual por concepto de insumos, materiales de protección y logístico, fue de 754,929.00 PEN; existiendo una gran diferencia en este rubro en comparación al año anterior, debido a que se realizaron más campañas; además, en el 2018 se efectuó la compra de gasolina de 90 octanos y se adquirió mayor cantidad de malatión, con un costo anual de 44,454.00 PEN. Los costos por equipos y transporte solo alcanzaron un costo anual del 60,181.25 PEN. Por último, el costo sanitario indirecto anual fue de 169,470.00 PEN (Tabla 2).

En cuanto al control vectorial de tipo larvario, esta se realiza en forma programada, en cinco campañas o ciclos de intervención de dos meses de duración cada ciclo. Dentro de estas actividades se efectúan actividades de vigilancia como encuestas aédicas, inspección de viviendas y uso de controladores o inhibidores del crecimiento larvario, además esto implica la eliminación de criaderos y orientación sobre prevención en los hogares. Se contó con 130 inspectores de viviendas, quienes se distribuyeron en los diferentes establecimientos de la región, 108 en la ciudad de Iquitos y 22 en la periferia; estos inspectores realizaron las encuestas aédicas y la aplicación de piriproxifen 0,5% para el control larvario.

En la Tabla 3, se muestra los costos de la intervención de inspección de viviendas con riesgo de transmisión del dengue y la aplicación de encuestas aédicas, esta se divide en costos sanitarios directos e indirectos; en cuanto a los costos sanitarios directos, este se dividió en personal que participa directamente en la inspección de viviendas y encuesta aédica, con un costo total para el 2017 de 2,025,600.00 PEN; personal de apoyo que labora en la oficina de control vectorial con un costo total de 83,160.00 PEN; luego están los insumos, materiales de protección y logísticos con un costo total de 549,185.00 PEN; dentro de estos se encuentra el insecticida piriproxifen 0,5% con un costo de 375,900.00 PEN. Por último, están otros gastos como capacitaciones con 7,225.00 PEN; y encuesta aédica con 134,888.50 PEN.

**Tabla 3:** Costo anual de las inspecciones de vivienda y encuesta aédica: costos sanitarios directos e indirectos. Dirección Regional de Salud Loreto, 2017 - 2018.

			2017	2018	Total (PEN)
			Costo (PEN)	Costo (PEN)	
Costos sanitarios directos					
	Personal profesional principal				
		Jefes de Brigada	580,800.00	660,000.00	1,240,800.00
		Inspectores	1,372,560.00	1,231,200.00	2,603,760.00
		Encuestas aédicas	72,240.00	64,800.00	137,040.00
		Total	2,025,600.00	1,956,000.00	3,981,600.00
	Personal de Apoyo				
		Biólogo	60,000.00	60,000.00	
		Director del centro de control vectorial	13,440.00	13,440.00	
		Técnico administrativo	9,720.00	9,720.00	
		Total	83,160.00	83,160.00	166,320.00
	Insumos y materiales				
		Piripoxifen 0,5%	375,900.00	105,000.00	
		Guantes	39,000.00	39,000.00	
		Mochila	32,500.00	32,500.00	
		Polos	7,800.00	7,800.00	
		Otros	31,336.50	42,751.50	
		Materiales para encuesta aédica	62,648.50	51,433.50	
		Total	549,185.00	278,485.00	827,670.00
	Otros gastos				
		Capacitaciones	7,225.00	7,225.00	14,450.00
		Encuestas aédicas	134,888.50	116,233.50	251,122.00
	Total costos sanitarios directos		2,665,170.00	2,324,870.00	4,990,040.00
Costos sanitarios indirectos					
	Personal de sede central DESA				
		Director ejecutivo	4,500.00	4,500.00	
		Administrador	3,500.00	3,500.00	
		Otros	4,200.00	4,200.00	
		Total	12,200.00	12,200.00	24,400.00
	Gastos de oficina y gastos varios				
		Material y equipos de oficina	5,000.00	5,000.00	
		Alquiler de local principal DESA	11,000.00	11,000.00	
		Otros	4,200.00	4,200.00	
		Total	20,200.00	20,200.00	40,400.00
	Total costos sanitarios indirectos		32,400.00	32,400.00	64,800.00
Costo total del control larvario			2,697,570.00	2,357,270.00	5,054,840.00

PEN: soles peruanos, DESA: Dirección ejecutiva de saneamiento ambiental, DIRESA: Dirección Regional de Salud Loreto.

En el 2018, el costo en personal inspector de vivienda fue de 1,956,000.00 PEN; el costo por personal de apoyo fue de 83,160.00 PEN; por insumos, materiales de protección personal y logísticos el costo fue de 278,485.00 PEN, dentro de este costo se incluye al piriproxifen al 0,5% con un costo de 105,000.00 PEN; con una diferencia sustancial si los comparamos con el año anterior. Por último, están otros gastos como capacitaciones a los inspectores con un costo de 7,225.00 PEN y encuesta aédica con 116,233.50 PEN.

En cuanto a los costos sanitarios indirectos, tanto para el 2017 y 2018 fue un total de 32,400.00 PEN por año.

Los casos de dengue se incrementaron estacionalmente en los dos años de estudio, de acuerdo a lo reportado por la Dirección de Epidemiología de la DIRESA Loreto.

## DISCUSIÓN

El costo total anual del control del *Aedes aegypti* en la región Loreto fue de 3,807,858.73 PEN (1,175,264 USD) para el 2017 y de 4,066,380.25 PEN (1,210,232 USD) para el 2018.

Las actividades de control vectorial en el Perú se realizan de acuerdo con la normatividad del MINSA ^9^. El control vectorial se realiza en los escenarios II y III (presencia del vector con casos esporádicos y presencia del vector en brotes). En el escenario II, el objetivo es reducir el riesgo de transmisión de dengue y se dirige a acciones de control del mosquito en su fase larvaria. En el escenario III, el objetivo es controlar rápidamente la transmisión y se aplican métodos de control del mosquito tanto en fase larvaria como en fase adulta.

Los costos hallados son altos comparados con lo reportado en otros países, considerando que Loreto tiene cerca de un millón de habitantes, sin embargo, en otros países donde se transmite el dengue como en Perú, se dedican importantes recursos al control del vector ^12^^-^^15^. Existen pocos estudios sobre costos en el Perú y estos no describen en detalle los gastos incurridos en el control vectorial. En Piura, en el 2002, se reportó un gasto de 64,260.00 PEN en 50 días en el control vectorial de un brote de dengue en la localidad de Sechura ^16^. Salmon-Mulanovich *et al.* en 2014, calcularon el costo de la atención de casos, en un brote de dengue en una región amazónica, hallando que el costo para cada caso era en promedio 105,3 USD ^7^. Stahl *et al*. calcularon en 4,5 millones USD el costo del brote del 2011 en Perú, del cual el 16% correspondía al control vectorial (738,701 USD) ^8^.

Los métodos de control vectorial usados serían eficaces para disminuir la densidad vectorial ^17^, el control vectorial programado (control larvario, insecticidas, biológicos, etc.), costaría menos que el control vectorial en respuesta a brotes. En el Perú se ha evaluado la efectividad del control vectorial, Stoddard *et al*. en un estudio que registró datos de una década en la ciudad de Iquitos, concluyen que existiría evidencia del impacto del control vectorial de adultos en la transmisión del dengue si es aplicada tempranamente en los brotes ^18^. Por otro lado, en Iquitos, Reiner *et al*. elaboraron un modelo basado en la densidad de adultos del *Aedes aegypti*, recolectado por varios años, que demuestra que, dependiendo de la cobertura de viviendas alcanzada en la nebulización espacial, se puede conseguir entre 67 a 43% en la reducción de la densidad de adultos hembras, responsables de la transmisión, si se logra el 100 o 50% de cobertura de viviendas intervenidas, respectivamente ^19^.

Los métodos usados serían efectivos para disminuir la densidad del vector, pero, su efecto es de corta duración y más costoso en el tiempo. Según Pontes *et al*. el control larvario tendría una persistencia de solo dos meses en el mejor de los casos ^20^. El control de adultos con la aplicación de insecticida de volumen ultrabajo tiene un efecto inmediato en la población de mosquitos, que duraría un solo día, según Koenraadt *et al.*^21^. Dado el corto efecto de los métodos usados, para mantener la densidad del *Aedes aegypti* en niveles bajos es necesario realizar periódicamente los ciclos de control larvario y el control de adultos, todo esto a un costo alto, no lográndose un efecto prolongado o control del vector definitivo con las técnicas usadas en la actualidad. Asimismo, debemos tener en cuenta que, en brotes con gran población afectada es, a veces, necesario incrementar la frecuencia de las intervenciones para el control de adultos. El 2011, de acuerdo con los entomólogos de la DIRESA Loreto, se hicieron más de 10 intervenciones para adultos en un año por la magnitud del brote. Por otro lado, el efecto de las intervenciones basadas en insecticidas disminuye en el tiempo debido a la emergencia de resistencia del vector, teniendo que reemplazarlos por moléculas de mayor costo y toxicidad ^1^.

Existirían otros condicionantes por donde es posible intervenir, por ejemplo, en Iquitos, como en otras ciudades del país, el suministro de agua es menor a cinco horas diarias, lo que obliga a la población a almacenar agua en todo tipo de recipientes que se convierten en criaderos del vector ^6^. Asimismo, la acumulación de inservibles y las lluvias frecuentes crean las condiciones para el crecimiento del vector.

Durante el 2017, en la costa norte peruana, se presentó uno de los brotes más grandes de dengue registrados por el MINSA en el Perú, se presentaron 68,290 casos y 89 fallecidos ^3^. En ese mismo año, en Loreto solo se presentó un incremento estacional de casos, talvez como efecto del control vectorial sistemático o del clima.

Un fenómeno observado recientemente es la invasión del *Aedes aegypti* a pequeñas comunidades rurales, probablemente por la adopción de costumbres que facilitan el desarrollo del vector, lo cual incrementaría los costos del control ^22^.

Una limitación de este estudio fue que el análisis del control del mosquito adulto (nebulización espacial con equipo portátil) se basó principalmente en datos de la ciudad de Iquitos, debido a que las intervenciones realizadas en otras ciudades de la Región Loreto son difíciles de cuantificar por el subregistro de actividades. Asimismo, no se consideraron los costos de agua y luz de otros locales alquilados por considerarlos no significantes en comparación a otros costos. En esta estimación se evaluó solo costos por lo que es una evaluación económica de tipo parcial ^23^, al no estimar los beneficios o resultados en la salud de la población, es posible, sin embargo, que este estudio pueda servir de línea de base para estudios posteriores que evalúen el impacto teniendo en cuenta que la pobre calidad de los datos y el subregistro existente en países endémicos dificultan el análisis del impacto del control vectorial ^24^. Debemos considerar que esta estimación se realizó en años con moderado número de casos y no en un año con intensa transmisión como lo ocurrido en el 2011 en Loreto, lo cual hubiera elevado los costos.

En conclusión, los costos incurridos en el control del vector del dengue en la Región Loreto en los dos años estudiados superan el millón de dólares, por año. Estas intervenciones tienen el efecto inmediato en reducir la transmisión, pero este efecto es corto y crean una dependencia al uso de insecticidas cada vez mas costosos.
